# The Protective Impact of *Salsola imbricata* Leaf Extract From Taif Against Acrylamide-Induced Hepatic Inflammation and Oxidative Damage: The Role of Antioxidants, Cytokines, and Apoptosis-Associated Genes

**DOI:** 10.3389/fvets.2021.817183

**Published:** 2022-01-28

**Authors:** Mohamed Mohamed Soliman, Saqer S. Alotaibi, Samy Sayed, Mohamed M. Hassan, Fayez Althobaiti, Adil Aldhahrani, Gehan B. A. Youssef, Ahmed M. El-Shehawi

**Affiliations:** ^1^Clinical Laboratory Sciences Department, Turabah University College, Taif University, Taif, Saudi Arabia; ^2^Department of Biotechnology, College of Science, Taif University, Taif, Saudi Arabia; ^3^Department of Science and Technology, University College-Ranyah, Taif University, Taif, Saudi Arabia; ^4^Department of Biology, College of Science, Taif University, Taif, Saudi Arabia; ^5^Forensic Medicine and Toxicology Department, Faculty of Veterinary Medicine, Benha University, Benha, Egypt

**Keywords:** *Salsola imbricata*, hepatic toxicity, acrylamide, gene expression, antioxidants, oxidative stress

## Abstract

*Salsola imbricata* is a herbal plant native to Saudi Arabia, known for its antioxidative and anti-inflammatory properties. This study explored the protective effects of an ethanolic leaf extract of *Salsola imbricata* against the oxidative stress and hepatic injury caused by acrylamide. Rats received intragastric administrations of 20 mg/kg of body weight of acrylamide to induce hepatic injury, or 300 mg/kg of body weight of *Salsola* ethanolic extract orally for 7 days before acrylamide administration. The treatments were continued for 3 weeks. Blood and liver samples were collected from all the groups, and the following biochemical parameters were tested: serum ALT (alanine aminotransferase), AST (aspartate aminotransferase), GGT (gamma glutaryl transferase), urea, albumin, total proteins, catalase, SOD (superoxide dismutase), reduced glutathione (GSH), nitric oxide (NO), and MDA (malondialdehyde). Quantitative real-time PCR (qRT-PCR) was used to examine the expression of Nrf2 (Nuclear factor-erythroid factor 2-related factor 2), HO-1 (Hemoxygenase-1), COX-2 (Cyclooxgenase-2), TGF-β1 (transforming growth factor-beta1), Bax, and Bcl2 (B-cell lymphoma 2), which are associated with oxidative stress, fibrosis, apoptosis, and anti-apoptotic effects. The annexin and survivin immunoreactivity were examined at the immunohistochemical level. Pretreatment with the *Salsola* ethanolic extract reduced the negative impact of acrylamide on ALT, AST, GGT, urea, albumin, and total proteins. The *Salsola* ethanolic extract reversed acrylamide's effects on serum and tissue antioxidants. Nrf2/HO-1 expression was downregulated, while COX-2 and TGF-β1 were upregulated in the acrylamide-administered group and normalized by the pre-administration of *Salsola* ethanolic extract to the acrylamide experimental group. The immunoreactivity of annexin and survivin was restored in the experimental group administered *Salsola* ethanolic extract plus acrylamide. In conclusion, *Salsola* ethanolic extract inhibits and regulates the side effects induced in the liver by acrylamide. *Salsola* induced its impacts by regulating inflammation, oxidative stress, and apoptosis-/anti-apoptosis-associated genes at the biochemical, molecular, and cellular levels. *Salsola* is recommended as oxidative stress relievers against environmental toixicity at high altitude areas.

## Introduction

Humans are exposed to a variety of molecules on a daily basis that might cause significant diseases, either directly or indirectly, through the production of reactive substances such as reactive oxygen species (ROS) (1). Free radicals that induce lipid peroxidation are the most common causes of cell damage and organ dysfunction (2). Liver illnesses are regarded as one of the world's most serious health issues (3), and despite their high frequency, morbidity, and mortality rates, their present medical care is deemed insufficient. No medication has yet demonstrated total efficacy in stopping liver disease progression (4). Furthermore, the newly developed medications used to treat chronic liver disease are frequently linked to a variety of side effects, some of which are unacceptable (5). As a result, medicinal plants, particularly those with a long history of usage, have long been regarded as the main source of new therapeutic medications that could aid in the treatment of liver diseases (6).

Acrylamide is a white solid powder that readily dissolves in ethanol, water, and other solvents. Acrylamide is used in the water treatment, textile, printing, and cosmetics industries (7, 8). Researchers have known since 2002 that, in carbohydrate-rich meals that include potatoes, bread, biscuits, and grains, the carbohydrates can be converted into acrylamide at high temperatures (9). Several investigations, *in vitro* and *in vivo*, have convincingly confirmed its neurotoxicity and mutagenicity in both humans and animals, and have shown that exposure to acrylamide in high doses promotes cancer in the testes and thyroid gland, as well as mammary fibroadenomas, in experimental rats (10, 11). Organ toxicity from acrylamide administration has been reported in the last decade. Liver hepatocyte necrosis, increased liver biomarker enzymes, inflammatory cell infiltration, and fat buildup have been reported in several studies (11, 12). Acrylamide has been found to cause substantial pathological alterations in the kidneys, including acute tubular degeneration, bleeding, constriction, and closure of Bowman's gap (13, 14). Based on a previous study (15), acrylamide intoxication causes hepatorenal injuries through lipid peroxidation, oxidative stress, oxidative DNA damage, and inflammatory responses. ROS attack cell membranes and induce damage in biomolecules such as lipids, DNA, and proteins. Similarly, other study (16) reported that acrylamide induced oxidative damage in IEC-6 cells through the generation of ROS and MDA, and decreased the activities of superoxide dismutase (SOD) and glutathione peroxidase (GSH-PX).

There are over 100 species of the genus *Salsola* from the family Chenopodiaceae, which can be found in the dry regions of Europe, Asia, and Africa (17). *Salsola imbricata Forssk* is one of these species, a saline and sandy-growing shrub that can be found in the Taif area. *Salsola imbricata* ha*s* other names such as Lani, Haram, and Lana. *Salsola* can be used to treat indigestion, diarrhea, dysentery, colds, asthma (18), and the congestion of the sinuses in folk medicine (19). The herb is also used in expelling parasitic worms (20), as an antioxidant, and as a diuretic (21). In several extracts of *Salsola imbricata*, phytochemical investigation has revealed the presence of tannins, anthraquinones, alkaloids, saponins, and flavonoids (22, 23). Coumarins have been identified from a *Salsola imbricata* methanolic extract (24). The primary phenolics in the plant's hydrolyzed ethanol extract have been identified as quercetin and coumaric acid (25). The phenolic and flavonoids compounds were highly concentrated in the leaf part of the plant compared to other parts; stem, root and bark parts (26, 27).

Oxidative injury mediated by free radicals due to living at high altitudes is an important factor in the pathogenesis of some diseases and in adverse metabolic reactions (28). Free radicals generated from oxidative stress due to living at high altitudes react with proteins, leading to their inactivation and the formation of carbonyls. These carbonyls may lead to functional impairment and complete cell and organ dysfunction, especially in the liver. Because liver diseases are a severe threat to human health, especially in high-altitude areas, the demand for a safe therapy is increased. Till now, no direct clear study showed the potential mechanism of *Salsola Imbricata* against hepatic toxicity. Only one study compared the effect of *S. imbricata* and other 3 medicinal plants against hepatic toxicity (17). As previously established, herbal medications are considered safe for human health compared to synthetic drugs that have more side effects. The usage of *Salsola imbricata* as a protective agent against organ toxicity induced by oxidative stress has not been postulated. Therefore, we examined the protective effect of an ethanolic extract of *Salsola imbricata* against hepatotoxicity. *Salsola imbricata* has not yet been investigated as a liver-protecting agent or as a new herbal remedy for treating organ toxicity.

## Materials and Methods

### Materials and Kits

Kits for the determination of aspartate aminotransferase (AST), alanine aminotransferase (ALT), and gamma glut-aryl transaminase (GGT) were obtained from Biomed Diagnostics Co, Giza, Egypt. Pure acrylamide (99%) was purchased from Sigma Aldrich (St Louis, MO, USA). ELISA kits for analyzing cytokines were purchased from R&D (Mannheim, Germany). Kits for antioxidants (MDA, SOD, GSH, NO and catalase), urea, albumin, and total proteins were bought from Bio-diagnostics Co. (Cairo, Egypt). Reverse transcriptase enzymes for RNA and other related markers were bought from MBI (Fermentas, Thermo Fisher Scientific, USA). Oligo dT and Qiazol were purchased from QIAGEN Company (Valencia, CA, USA). Primers were designed and ordered from Macrogen CO. (Seoul, Korea).

### Animals

This study used 40 10-week-old male rats. The rats had free access to water and food and were housed at room temperature. The rats were handled manually for 7 days in the labs of Turabah University so that they became totally adapted to human contact. The animals were handled a cording to the guidelines used in Taif University for the project number 136-441-1, September 11, 2020.

### *Salsola imbricata* Leaf Extraction

Fresh leaves of *Salsola imbricata* plants were collected in October 2020 from the Al-Hada region, Taif Governorate, Saudi Arabia. The plant was identified by Botanist from Taif University, College of Science, Botany Department. The plant fresh leaves were air-dried and ground into a fine powder. Then, 300 grams of the fine powder was extracted with 300 mL of 95% ethanol at room temperature for 48 h. The obtained extract was then centrifuged at 9,000 × g for 7 min and filtered using Whatman paper1 (29). The supernatant layer was evaporated by passing it through a Buchner funnel in a rotavapor at 40°C. The residue was weighted and kept at −20°C for the *in vivo* experiments. For the total phenols and flavonoids, the residue was dissolved in dimethylsulfoxide (1% DMSO) and then used for HPLC analysis.

### Experimental Design and Sampling

**Figure 1:**
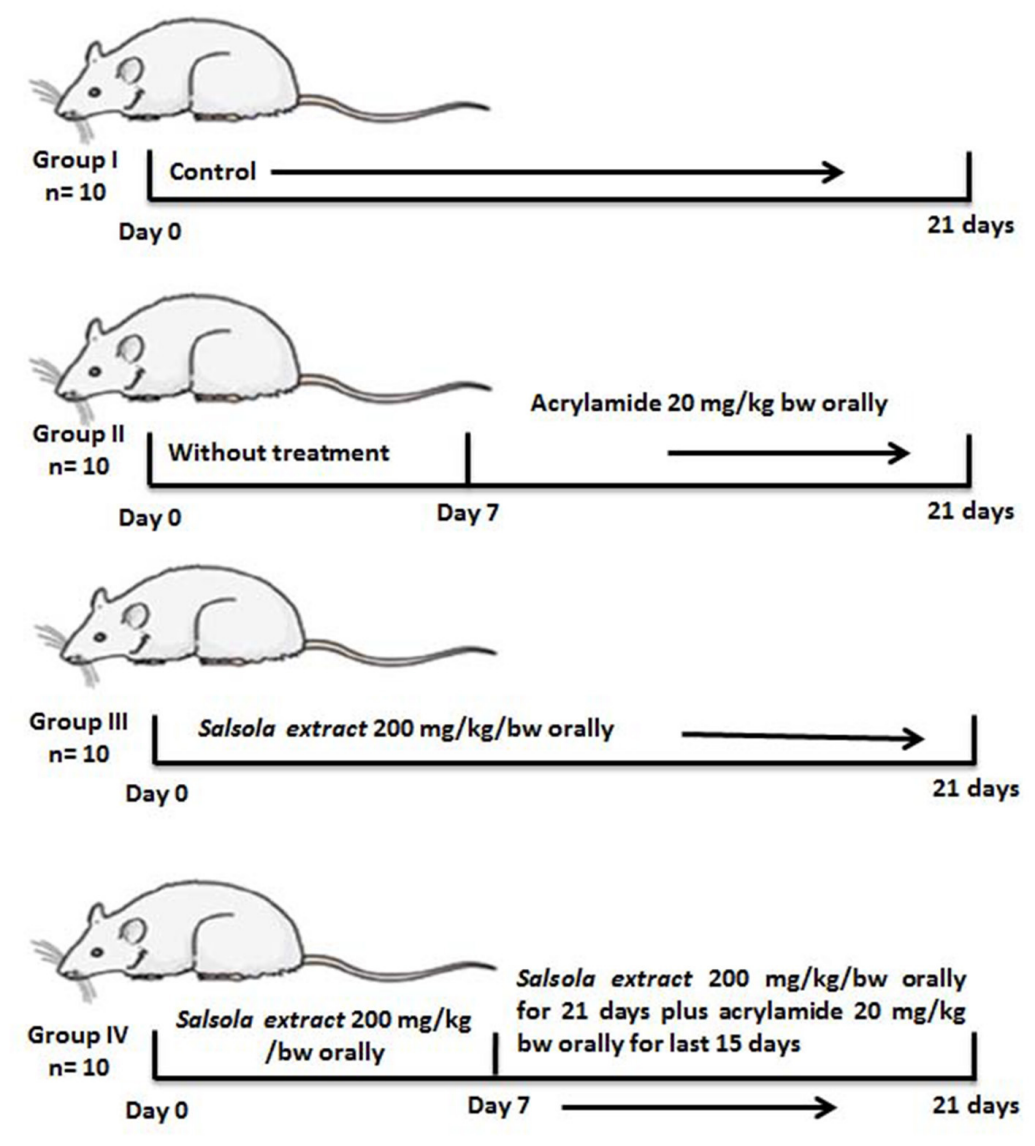
A schematic diagram for the experimental design used in the current study.

Four groups were separated out (10 rats per each group): Group 1, a negative control group received saline; group 2, an acrylamide-positive group, who orally received acrylamide at a dose of 20 mg/kg of BW once a day in saline (1, 15), this dose is optimal for inducing total toxicity of the liver (8); group 3, the *Salsola* group, orally administered *Salsola* extract at a dose of 200 mg/kg of BW once a day for 3 weeks in saline (17, 25); group 4, a protective group that received *Salsola* and acrylamide as stated in groups 2 and 3, with *Salsola* extract once a day, 1 week earlier than acrylamide. A schematic illustration of the experiments and design is shown in Figure 1.

On day 21, the experimental animals were anesthetized by isoflurane inhalation and decapitated. Blood samples and the liver tissues were harvested under aseptic conditions. The extracted serum was used for biochemical measurements, and stored at −20°C. Hepatic tissue (50 mg) was stored in QIAZOL reagent for RNA analysis and real-time PCR. Bouin's solution was used to preserve the liver samples for histological (H&E) and immunohistochemical staining.

### HPLC Analysis for Phenol and Flavonoid Compounds in the *Salsola imbricata* Extract

To analyze and detect the phenol and flavonoid compounds in the *Salsola* extract, a specific protocol was followed (30), with little modification, using the Agilent 1260 Infinity HPLC Series (Agilent, Santa Clara, CA 95051, USA), equipped with a quaternary pump. Kinetex® 5 μm EVO C18 100 × 4.6 mm (Phenomenex, Torrance, CA 90501-1430, USA) was used as the column and was operated at 30°C (31). The separation method was carried out using a ternary linear elution gradient with (A) 2% HPLC-grade water and H_3_PO_4_ (v/v; 1:1), (B) methanol, and (C) acetonitrile. Then, a final volume of 20 μL was injected. For the detection of phenols and flavonoids, an AVWD detector set at 284 nm was used.

### Chemistry Measurements

The serum levels of ALT, AST, GGT, and urea were measured using a colorimetric spectrophotometer, as described in the instruction manual. Procedures described elsewhere (32) were used to quantify malondialdehyde (MDA). The SOD, NO, and catalase activity were measured using previously established methods (33, 34), as were the albumin levels (35) and total proteins via the Lowry method (36). A spectrophotometer set at 412 nm was used to detect GSH according to the Tietze method (37).

### Measurement of Inflammatory and Anti-inflammatory Cytokines

ELISA kits for rat IL-1β and TNF-α (ab100768 and ab46070, respectively) were used with an ELISA spectrophotometer, as described in the instruction manual for each kit. IL-10 was measured using a commercial kit available from Abcam Co., Waltham, MA 02453, USA (Rat IL-10 ELISA Kit; ab100765). The levels were calculated based on data obtained from the ELISA reader according to the instructions provided for each parameter.

### Quantification of Genes by Quantitative Real-Time PCR

Total RNA was isolated from the liver samples and quantified using a spectrophotometer at 260 nm following the device's instructions (BIORAD, California 94547, USA). First, 2 μg of isolated RNA was used to make cDNA (MyTaq Red Mix, Bioline, Memphis, Tennessee 38134-5611). Using the SYBR Green master mix (Thermo Scientific, Waltham, MA, USA), the synthesized cDNA was amplified to quantify different hepatic genes. The primers used in this investigation are listed in Table 1. The data obtained using CFX96 Touch™ Real-Time PCR (BIORAD Co., California 94547, USA) in this study were analyzed via the ^2−ΔΔ^Ct method. The changes in the intensity and expression of the examined genes were measured using the comparative cycle threshold (CT) values, which were standardized to beta-actin.

**Table 1:** Primer sequences used for quantitative real time PCR in liver of rats.

**Gene**	**Accession number**	**Direction**	**Primer sequence**
TGF-b1	NM_021578.2	Sense	GGACTACTACGCCAAAGAAG
		Antisense	TCAAAAGACAGCCACTCAGG
COX2	NM 017232	Sense	TGATCTACCCTCCCCACGTC
		Antisense	ACACACTCTGTTGTGCTCCC
BAX	NM 017059	Sense	AGGACGCATCCACCAAGAAG
		Antisense	CAGTTGAAGTTGCCGTCTGC
Nrf2	NM_031789.2	Sense	TTGTAGATGACCATGAGTCGC
		Antisense	TGTCCTGCTGTATGCTGCTT
HO-1	NM_012580.2	Sense	GTAAATGCAGTGTTGGCCCC
		Antisense	ATGTGCCAGGCATCTCCTTC
SOD	NM_053425.1	Sense	ACACCTATGCACTCCACAGAC
		Antisense	ACATTCGACCTCTGGGGGTA
Catalase	NM_012520.2	Sense	GCGGGAACCCAATAGGAGAT
		Antisense	CAGGTTAGGTGTGAGGGACA
Bcl2	NM_016993	Sense	ACTCTTCAGGGATGGGGTGA
		Antisense	TGACATCTCCCTGTTGACGC
β-actin	NM 031144	Sense	AGGAGTACGATGAGTCCGGC
		Antisense	CGCAGCTCAGTAACAGTCCG

### Histopathology and Immunohistochemistry

For histopathology, the liver samples were first fixed in 10% neutral buffered formalin. Then, the fixed samples were stained with H&E (hematoxylin and eosin), as described previously (38). Immunohistochemical staining was performed elsewhere (39). The liver tissue sections on slides were rinsed in 0.05 M citrate buffer, pH 6.8. Non-specific binding on the stained slides was blocked by treating the sections with 0.3% H_2_O_2_ and a protein block. Then, the sections were incubated with primary antibody bound with rabbit polyclonal anti-annexin or anti-survivin (Novus Biologicals) at a 1:200 dilution. Next, the slides were washed three times in phosphate-buffered saline and then incubated with goat anti-rabbit secondary antibody (EnVision System Horseradish Peroxidase Labeled Polymer; Dako) for 40 min at room temperature. Following this, the slides were visualized with a DAB kit and then counterstained with Mayer's hematoxylin. The immunoreactivity indices of annexin and survivin are presented as percentages of the positive expression in a total of 1,000 cells/8 high microscopic power fields. The annexin and survivin immunostaining was determined as the positive expression area, which was detected using the ImageJ software (NIH).

### Statistical Analysis

The data are expressed as the means with standard errors of the means (SEMs). The current data were analyzed using the SPSS software for Windows with one-way ANOVA and Dunnett's *post-hoc* descriptive tests (SPSS, IBM, Chicago, IL, USA). Values with *p* < 0.05 were considered statistically significant compared to either control or acrylamide intoxicated groups.

## Results

### Total Phenol and Flavonoid Contents

**Figure 2:**
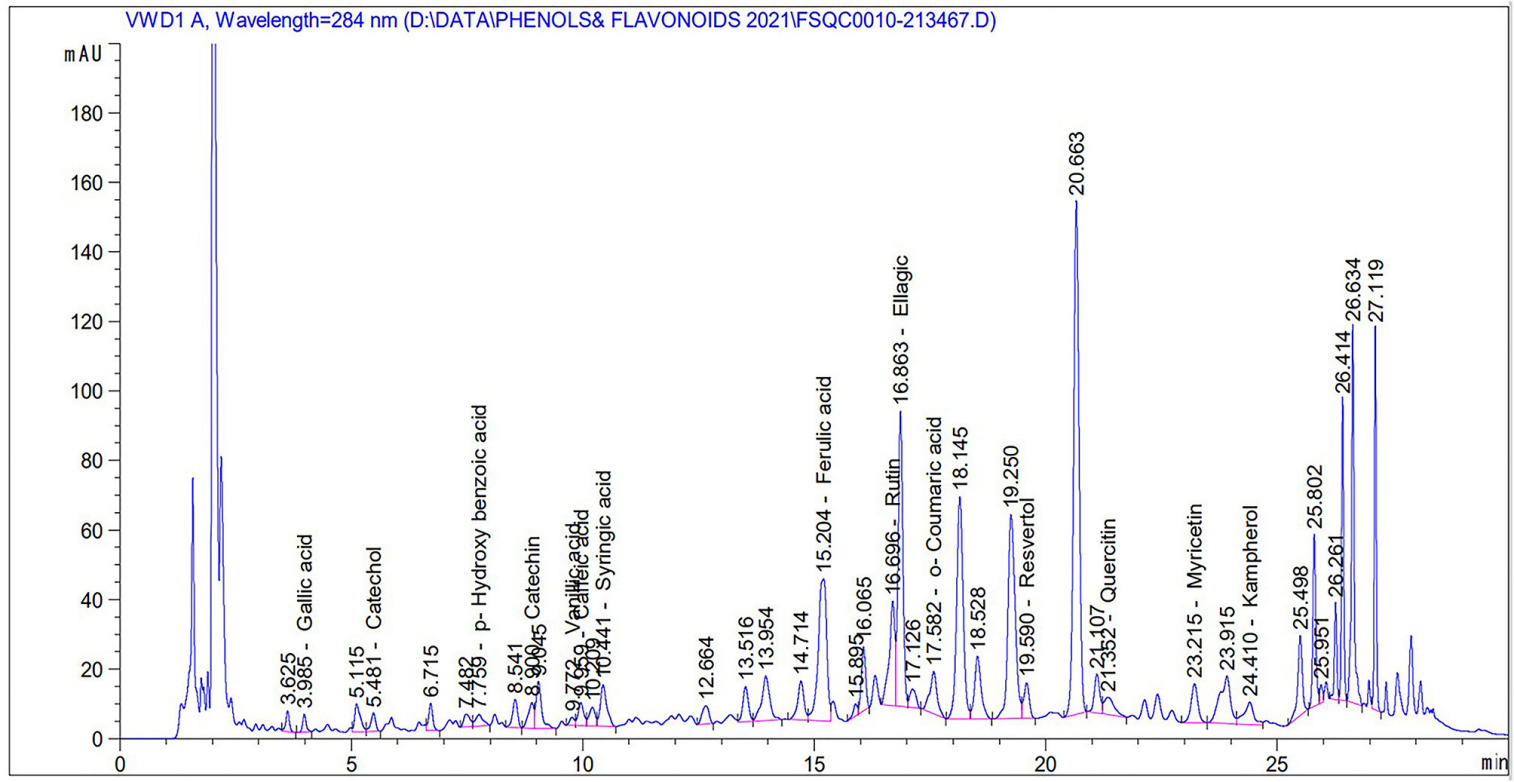
Total phenols and flavonoids of an ethanolic extract of *Salsola imbricata*.

Table 2 and Figure 2 show the phenol and flavonoid peaks and contents in the *Salsola* extract, respectively. The extract shows major contents of catechin (9.6 mg/L), vanillic acid (5.4/mg/L), catechol (4.26 mg/L), and p-hydroxy benzoic acid (3.79 mg/L). Other contents were detected as shown in Table 2, such as caffeic acid, syringic acid, ferulic acid, rutin, ellagic acid, o-coumaric acid, resveratrol, quercetin, myricetin, and kaempferol in different concentrations.

**Table 2:** Total phenols and flavonoids contents in ethanolic extract of *Salsola imbricata*.

**Name**	**Retention time**	**Area mAU**	**Amount (mg/L)**
Catechol	5.48	38.2	4.26
p-Hydroxy benzoic acid	7.759	36.6	3.79
Catechin	8.9	61.5	9.64
Vanillic acid	9.77	16.3	5.4
Caffeic acid	9.95	57.1	1.16
Syringic acid	10.4	115.9	3.36
Ferulic acid	15.2	522.3	1.38
Rutin	16.69	256.6	4.78
Ellagic	16.8	611.7	2.54
o-Coumaric acid	17.58	134.1	2.13
Resveratrol	19.59	84.4	2.23
Quercitin	21.35	74.55	2.38
Myricetin	23.2	111.8	3.79
Kaempferol	24.4	98.4	2.48

### Protective Impact of the *Salsola* Extract on Acrylamide-Induced Liver Dysfunction in Rats

Table 3 shows the elevation in serum biomarkers that reflect liver activity, such as AST, ALT, and GGT (*p* < 0.05). In parallel, there was a clear increase (*p* < 0.05) in urea levels, total proteins, and albumin after acrylamide administration. Liver toxicity was clear in the acrylamide-treated group, which was ameliorated in the protective group that received the *Salsola* extract for 1 week and was then given both *Salsola* and acrylamide for 2 weeks.

**Table 3:** Protective impacts of *Salsola* extract on acrylamide-induced liver dysfunction in rats.

	**Control**	**Acrylamide**	**Salsola**	**Salsola + Acrylamide**
AST (U/l)	25.7 ± 1.7	270.5 ± 18.1^*^	30.9 ± 1.8	80.9 ± 5.7^$^
ALT (U/l)	31.6 ± 1.3	206.8 ± 4.8^*^	31.1 ± 1.7	101.3 ± 3.9^$^
GGT (U/l)	4.2 ± 0.2	2.3 ± 0.3^*^	4.7 ± 0.3	3.7 ± 0.3^$^
Urea (mg/dl)	20.8 ± 1.1	45.4 ± 1.01^*^	20.9 ± 0.9	30.7 ± 1.2^$^
Albumin (g/dl)	6.3 ± 0.2	2.5 ± 0.1^*^	6.2 ± 0.2	4.8 ± 0.2^$^
Total proteins (g/dl)	10.3 ± 0.28	4.5 ± 0.19^*^	11.3 ± 0.48	7.3 ± 0.3^$^

*Values are means ± SEM for 10 different rats per each experiment. Values are statistically significant at*

* *p < 0.05 vs. Control and Salsola groups and*

$ *P < 0.05 vs. acrylamide group*.

### Impact of the *Salsola* Extract Against Acrylamide-Induced Changes in Antioxidants and the Biomarkers of Oxidative Stress

Table 4 shows the changes in the antioxidant levels. There was a significant decrease (*p* < 0.05) in the activities of SOD, GSH, and catalase in the sera of the acrylamide-administered rats. The levels of the examined antioxidants were decreased relative to those in the control and *Salsola-*extract-administered groups. The pre-administration of *Salsola* to the acrylamide group resulted in a recovery of antioxidants to normal levels in the protective group compared to salsola and acrylamide intoxicated rats. In contrast to the results for antioxidants, the levels of MDA in the acrylamide-injected rats were increased compared to those in the control and *Salsola*-administered groups. The pre-administration of *Salsola* to the acrylamide group protected the rats against the changes in MDA levels. The acrylamide-administered rats showed a significant increase in NO (*P* < 0.05). The prior administration of *Salsola* extract protected the rats (*p* < 0.05) from this increase compared to the acrylamide-administered rats.

**Table 4:** Protective effects of *Salsola extract* against acrylamide induced alterations on serum MDA, catalase GSH and NO and SOD levels.

	**MDA (nmol/ml)**	**Catalase (U/ml)**	**SOD (U/ml)**	**GSH (U/ml)**	**NO (nmol/ml)**
Control	10.4 ± 0.3	3.9 ± 0.1	26.89 ± 1.12	43.2 ± 1.4	34.5 ± 2.2
Acrylamide	24.5 ± 0.8^*^	1.5 ± 0.01^*^	13.09 ± 0.3^*^	19.2 ± 0.9^*^	169.6 ± 4.4^*^
Salsola	11.5 ± 0.6	3.5 ± 0.1	33.8 ± 1.7	42.8 ± 1.2	30.6 ± 1.6
Salsola + Acylamide	18.2 ± 0.2^$^	3.01 ± 0.1^$^	25.2 ± 0.8^$^	34.8 ± 1.5^$^	87.3 ± 2.3^$^

*Values are means ± standard error (SEM) for 10 different rats per each treatment. Values are statistically significant at*

* *P < 0.05 vs. Control and Salsola and*

$ *P < 0.05 vs. acrylamide group*.

### Impact of the *Salsola* Extract Against Acrylamide-Induced Changes in Inflammatory Cytokines (IL-1β and TNF-α) and Anti-inflammatory Cytokines (IL-10)

The administration of acrylamide significantly increased the serum levels of TNF-α and IL-1β (Table 5). *Salsola* administration alone resulted in a decrease (*p* < 0.05) in TNF-α and IL-1β levels. The prior administration of *Salsola* for a week and then for 2 weeks alongside acrylamide maintained the levels of the examined inflammatory cytokines around the control levels compared to those in the control and acrylamide received groups (Table 5). The acrylamide-administered rats showed a decline in the levels of the anti-inflammatory cytokine IL-10 compared to the control. *Salsola* increased IL-10 levels (*p* < 0.05) when given alone or before acrylamide administration. *Salsola* protected the rats from a decrease in IL-10 levels reported in the acrylamide-administered group (Table 5).

**Table 5:** Protective effects of *Salsola* against acrylamide-induced alterations in cytokines levels.

	**Control**	**Acrylamide**	**Salsola**	**Salsola + Acrylamide**
IL-1β	213.4 ± 9.7	558.2 ± 58.7^*^	168.0 ± 10.1^$^	194.8 ± 8.8^#^
TNF-α	300.6 ± 8.6	590.4 ± 26.2^*^	227.8 ± 15.6^$^	285.7 ± 9.7^#^
IL-10	116.7 5.6	54.1 ± 6.7^*^	132.8 ± 8.7^$^	95.9 ± 2.5^#^

*Values are means ± SEM for 10 different mice per each experiment. Values are statistically significant at;*

* *p < 0.05 vs. control and Salsola groups*,

$ *p < 0.05 vs. control and*

# *P < 0.05 vs. acrylamide group*.

### Boosting Impact of *Salsola* on the Expression of Antioxidants Genes

**Figure 3:**
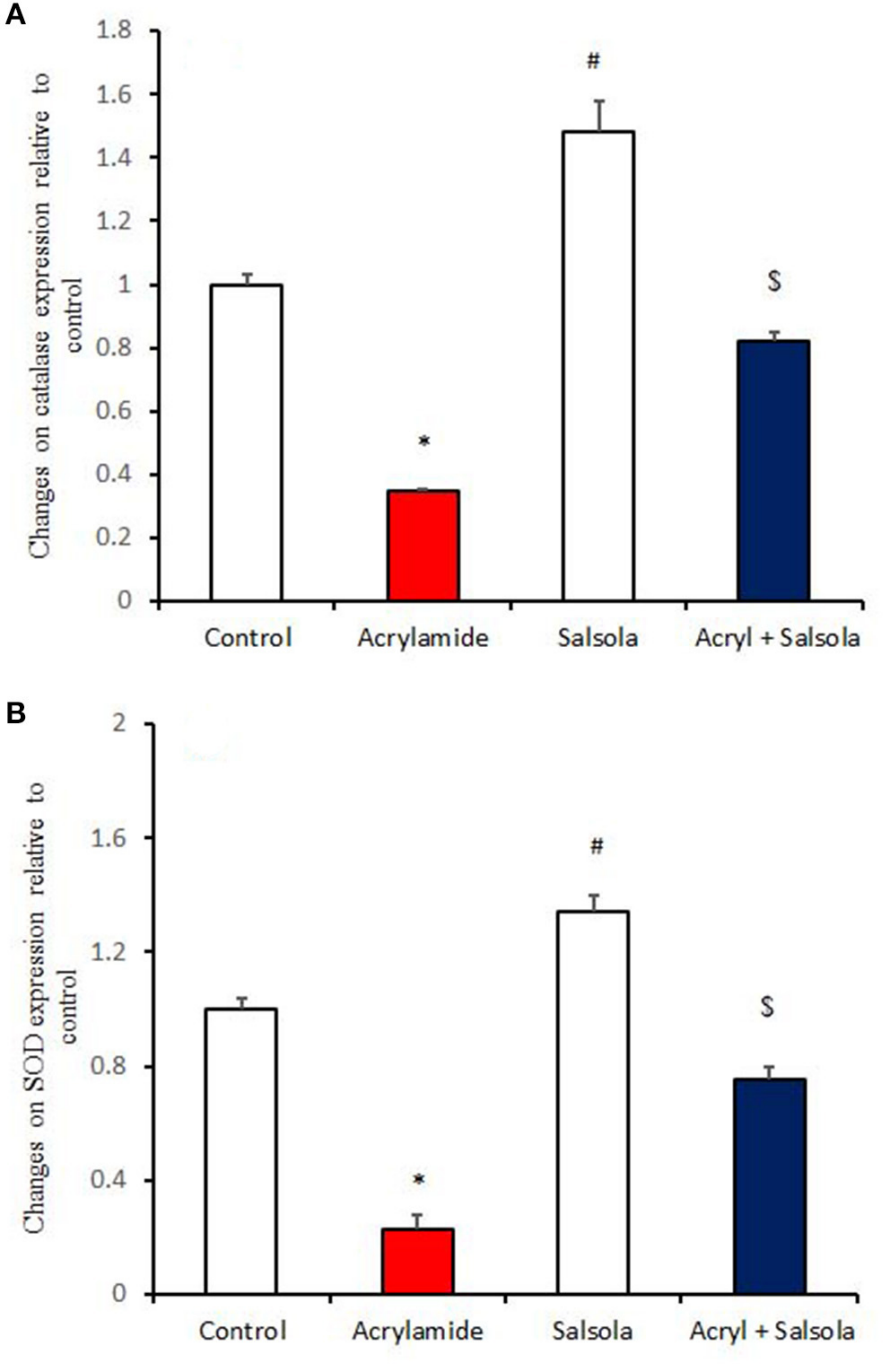
The ameliorative impact of *Salsola imbricata* extract on the mRNA expression of catalase and SOD in acrylamide-administered rats. Graphic presentation of hepatic expression according to qRT-PCR analysis of catalase and SOD in different groups of rats after normalization with the housekeeping gene (actin). *p < 0.05 vs. the control group; ^#^*p* < 0.05 vs. the other groups; ^$^*p* < 0.05 vs. the acrylamide-administered group.

Catalase and SOD expression was downregulated (*p* < 0.05) in liver tissue after acrylamide administration (Figures 3A,B), compared to that in the control and *Salsola*-administered groups. *Salsola* alone resulted in significant (*p* < 0.05) upregulation of catalase and SOD mRNA expression. The antioxidant activity in the liver was restored (*p* < 0.05) when *Salsola* was pre-administered to rats that received acrylamide, as both catalase and SOD showed normal expression patterns compared to acrylamide group (Figures 3A,B).

### Boosting the Impact of *Salsola* on Hepatic Oxidative Stress

**Figure 4:**
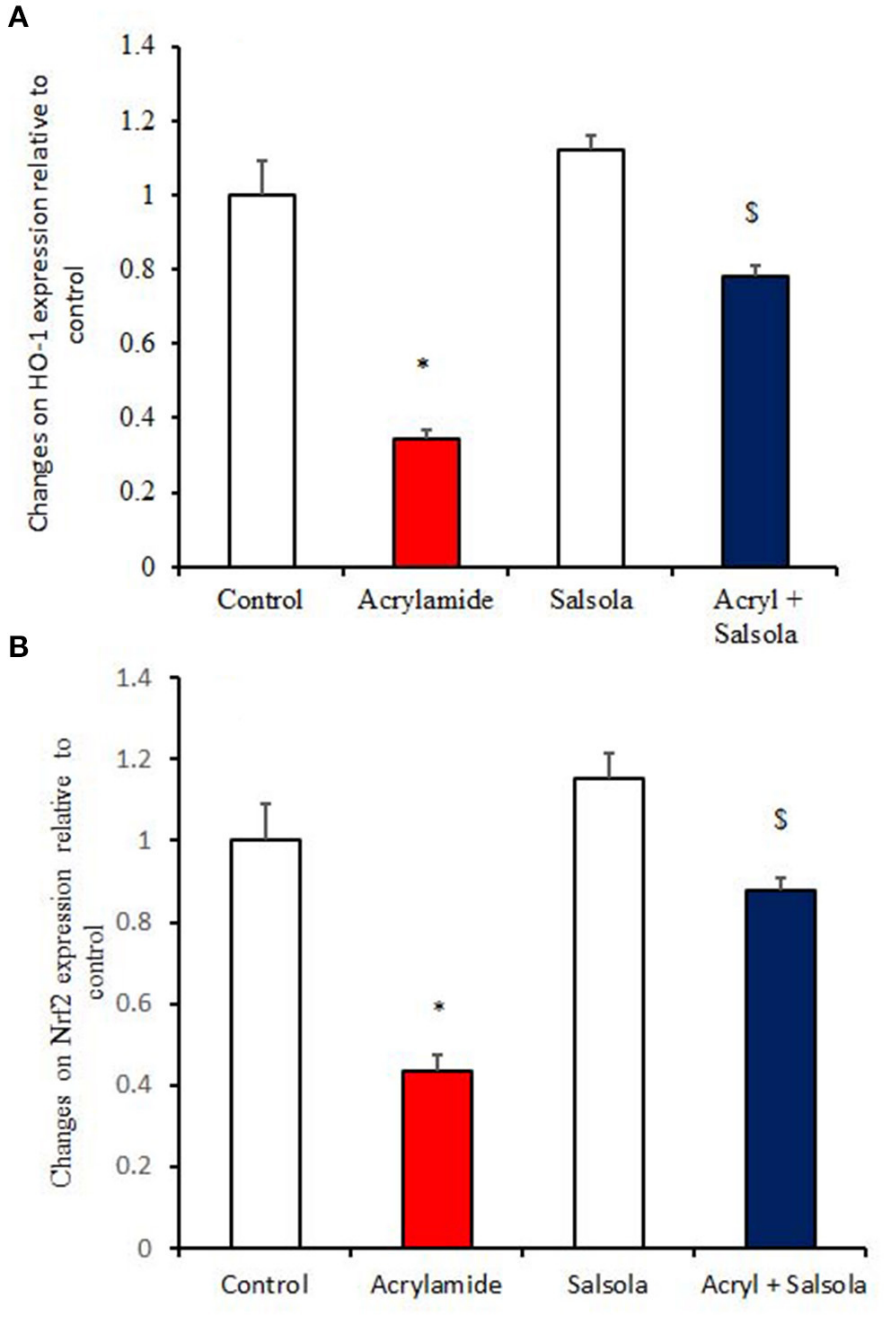
The ameliorative impact of *Salsola imbricata* extract on the mRNA expression of HO-1 and Nrf2 in acrylamide-administered rats (*n* = 10). Graphic presentation of hepatic expression by qRT-PCR analysis of HO-1 and Nrf2 in different groups of rats after normalization with the housekeeping gene (actin). *p < 0.05 vs. the control group; ^$^*p* < 0.05 vs. the acrylamide-administered group.

HO-1 and Nrf2 expression was downregulated (*p* < 0.05) in liver tissue after acrylamide administration (Figures 4A,B) in the acrylamide-administered rats, relative to that in the control and *Salsola* groups. The *Salsola* extract alone resulted in a noticeable upregulation of HO-1 and Nrf-2 expression. The liver antioxidant activity appeared to be restored (*p* < 0.05) when the *Salsola* extract was pre-administered to rats that received acrylamide, as both Nrf-2 and HO-1 showed normal expression patterns in the protective group (Figures 4A,B).

### Impact of the *Salsola* Extract on the Expression of Liver Fibrotic Genes

**Figure 5:**
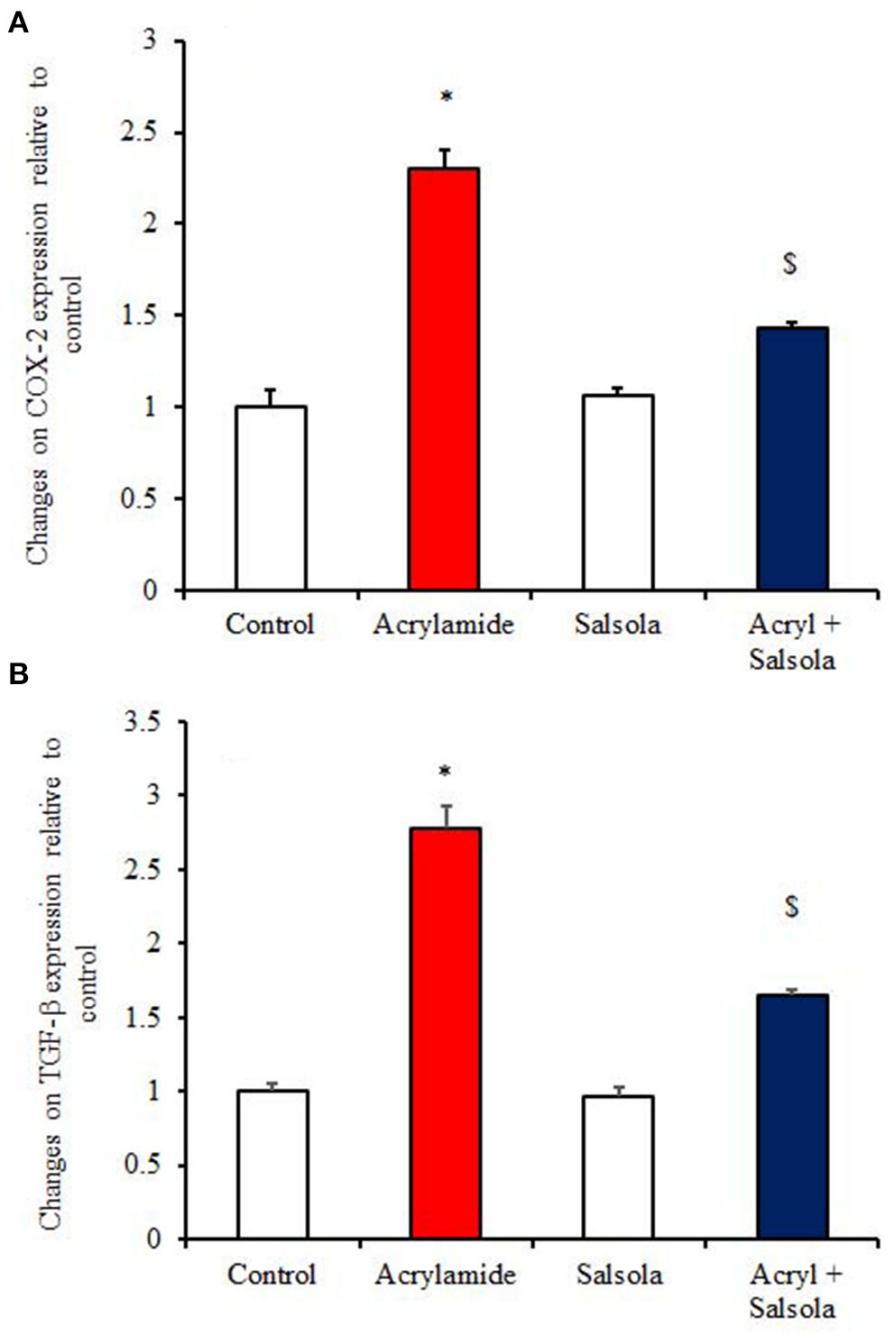
The impact of *Salsola imbricata* extract on the mRNA expression of COX-2 and TGF-β1 in acrylamide-administered rats (*n* = 10). Graphic presentation of hepatic expression according to qRT-PCR analysis of COX-2 and TGF-β1 in different groups of rats after normalization with the housekeeping gene (actin). *p < 0.05 vs. the control group; ^$^*p* < 0.05 vs. the acrylamide-administered group.

The acrylamide-administered group showed upregulation (*p* < 0.05) of the mRNA expression of COX-2 and TGF-β1 (Figures 5A,B), while the *Salsola*-plus-acrylamide group showed downregulated COX-2 and TGF-β1 expression. Compared to the control and acrylamide-administered groups, *Salsola* resulted in a significant (*p* < 0.05) recovery and restoration for the altered genes.

### Boosting the Impacts of *Salsola* on the Expression of Apoptotic and Anti-apoptotic Genes

**Figure 6:**
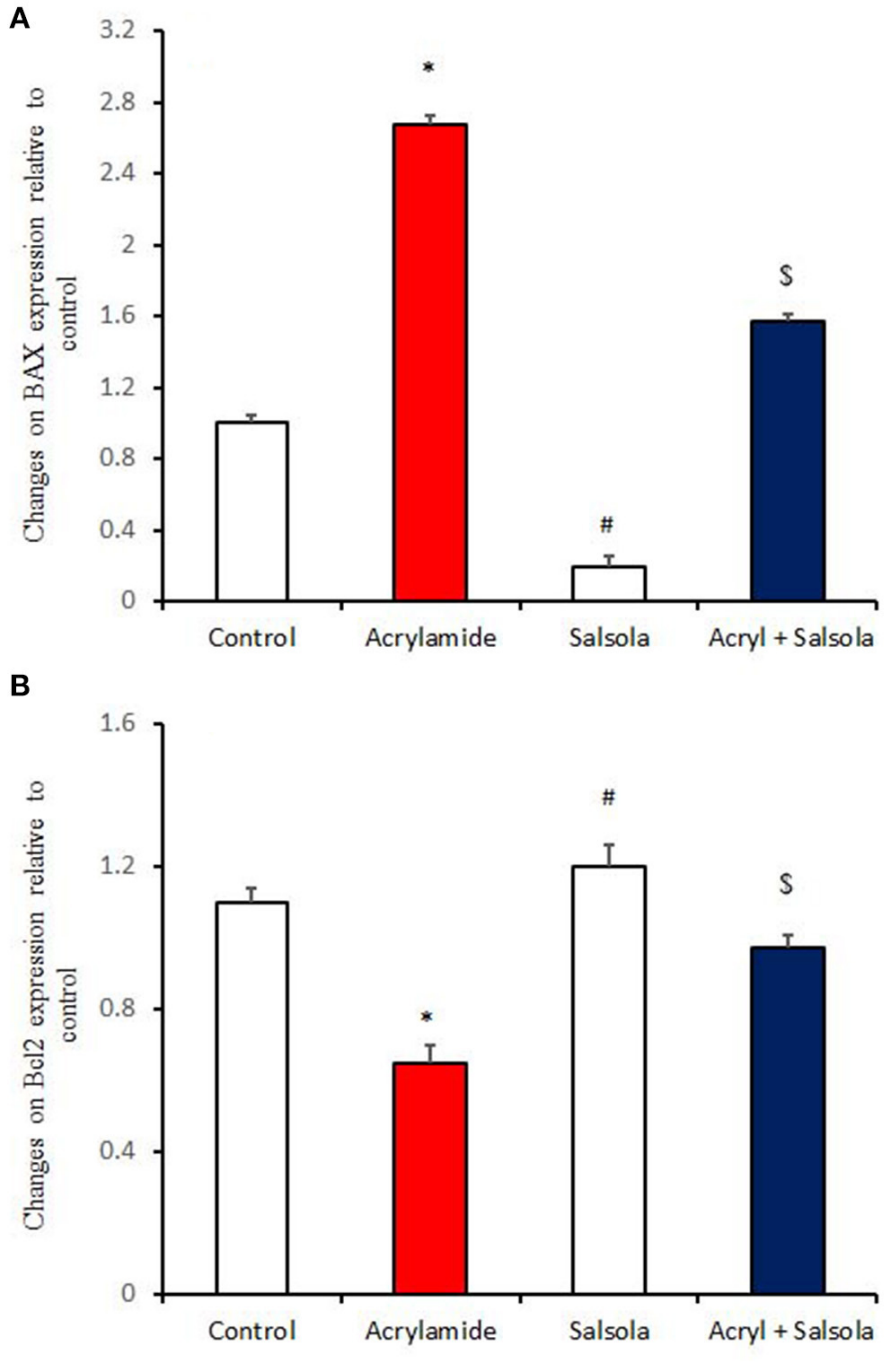
The impact of *Salsola imbricata* extract on the mRNA expression of Bax and Bcl2 in acrylamide-administered rats (*n* = 10). Graphic presentation of hepatic expression according to qRT-PCR analysis of Bax and Bcl2 in different groups of rats after normalization with the housekeeping gene (actin). *p < 0.05 vs. the control group; ^#^*p* < 0.05 vs. the other groups; ^$^*p* < 0.05 vs. the acrylamide-administered group.

BAX expression was upregulated (*p* < 0.05) in liver tissue after acrylamide administration (Figure 6A), indicating a general state of apoptosis in the acrylamide-administered rats, compared to the control and *Salsola-*extract-administered groups (*p* < 0.05). By contrast, Bcl2 showed downregulation (*p* < 0.05) in the acrylamide-treated rats, confirming the apoptotic effect of acrylamide. *Salsola* alone significantly upregulated (*p* < 0.05) Bcl2 and downregulated BAX expression. When *Salsola* was re-administered to the acrylamide-treated rats and continued for 15 days, it restored the alterations reported in the expression of both BAX and Bcl2 expression in acrylamide group (Figures 6A,B).

### Histopathological and Immunohistochemical Findings

**Figure 7:**
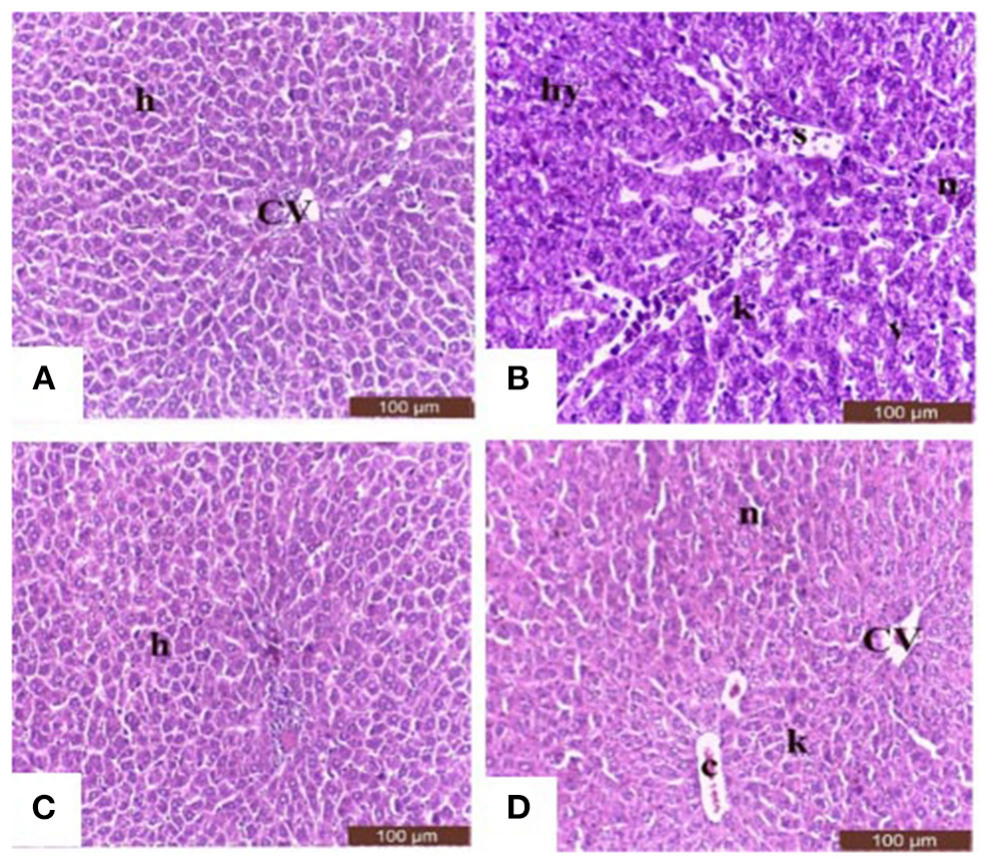
Photomicrographs of hepatic sections stained with hematoxylin and eosin in the control group **(A)**, the *Salsola*-treated group **(C)**, the acrylamide-treated group **(B)**, and the group co-treated with acrylamide and *Salsola* **(D)**. Sections from control and *Salsola extract* groups **(A,C)** showed the liver consisted of central vein (CV) surrounded by hepatic cords (h). The cords consisted of large hepatocytes with centrally located nuclei and acidophilic cytoplasm. Sections from the acrylamide-treated rats **(B)** the liver tissue showed vacular degeneration (v), hydrobic degeneration (hy). The liver tissues showed proliferation of the vonkupher cells (k). The blood sinusoids showed lymphocytic infiltrates (s). Some heaptocytes cells were characterized by necrosis (n) table (6). Co-treatment of rats with acrylamide and *Salsola* **(D)** both of the vacuolar and the hydrobic degeneration were localized to few hepatocyte cells. Some cells still in necrotic pattern (n). The vonkupher cells still proliferated but not numerous compared with acrylamide group. Scale bar = 20 μm (original magnification = 200 ×). The morphometric analysis and scoring of *Salsola imbricata extract* against acrylamide toxicity were shown on Table 6. Scale bar = 20 μm (original magnification = 200 ×).

Sections from control and Salsola administered rats (Figures 7A,C) showed the liver consisted of central vein (CV) surrounded by hepatic cords (h). The cords consisted of large hepatocytes with centrally located nuclei and acidophilic cytoplasm. Sections from acrylamide-treated rats (Figure 7B) showed showed that vascular degeneration (v) and hydrobic degeneration (hy) of hepatocytes. The liver tissues showed proliferation of the vonkupher cells (k). The blood sinusoids showed lymphocytic infiltrates (s). Some heaptocytes cells were characterized by necrosis (n). The co-treatment of rats with acrylamide and *Salsola* (Figure 7D) showed both of the vacuolar and the hydrobic degeneration were localized to few hepatocyte cells. Some cells still in necrotic pattern (n). The Kupffer cells still proliferated but not numerous compared with acrylamide group.

**Figure 8:**
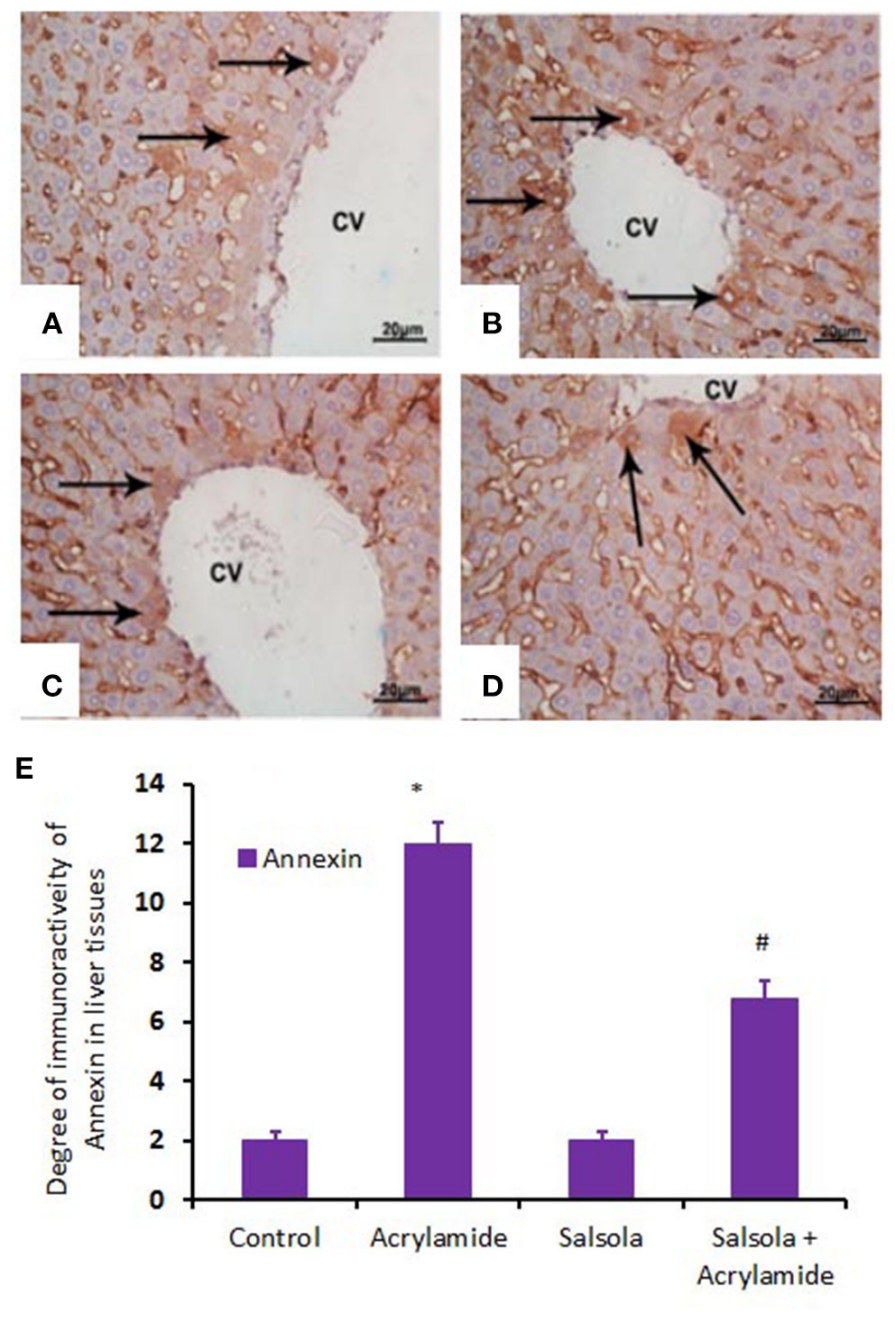
Photomicrographs of hepatic sections immunostained with annexin antibody in the control group **(A)**, the *Salsola*-treated group **(C)**, the acrylamide-treated group **(B)**, and the group co-treated with *Salsola* and acrylamide **(D)**. The intensity of immunostaining was weak in the control and *Salsola*-treated groups **(A,C)**, strong in the acrylamide-treated group **(B)**, and reduced to weak or moderate in the co-treated rats **(D)**. Reactive liver cells (arrows) are frequently seen next to central veins (CVs). Scale bar = 20 μm (original magnification = 200 ×). The degree of positive immunoreactivity for annexin is graphed in **(E)**. Densitometric values are statistically significant at *p < 0.05 vs. the control and *Salsola*-treated groups; ^#^*p* < 0.05 vs. the acrylamide-administered group.

**Figure 9:**
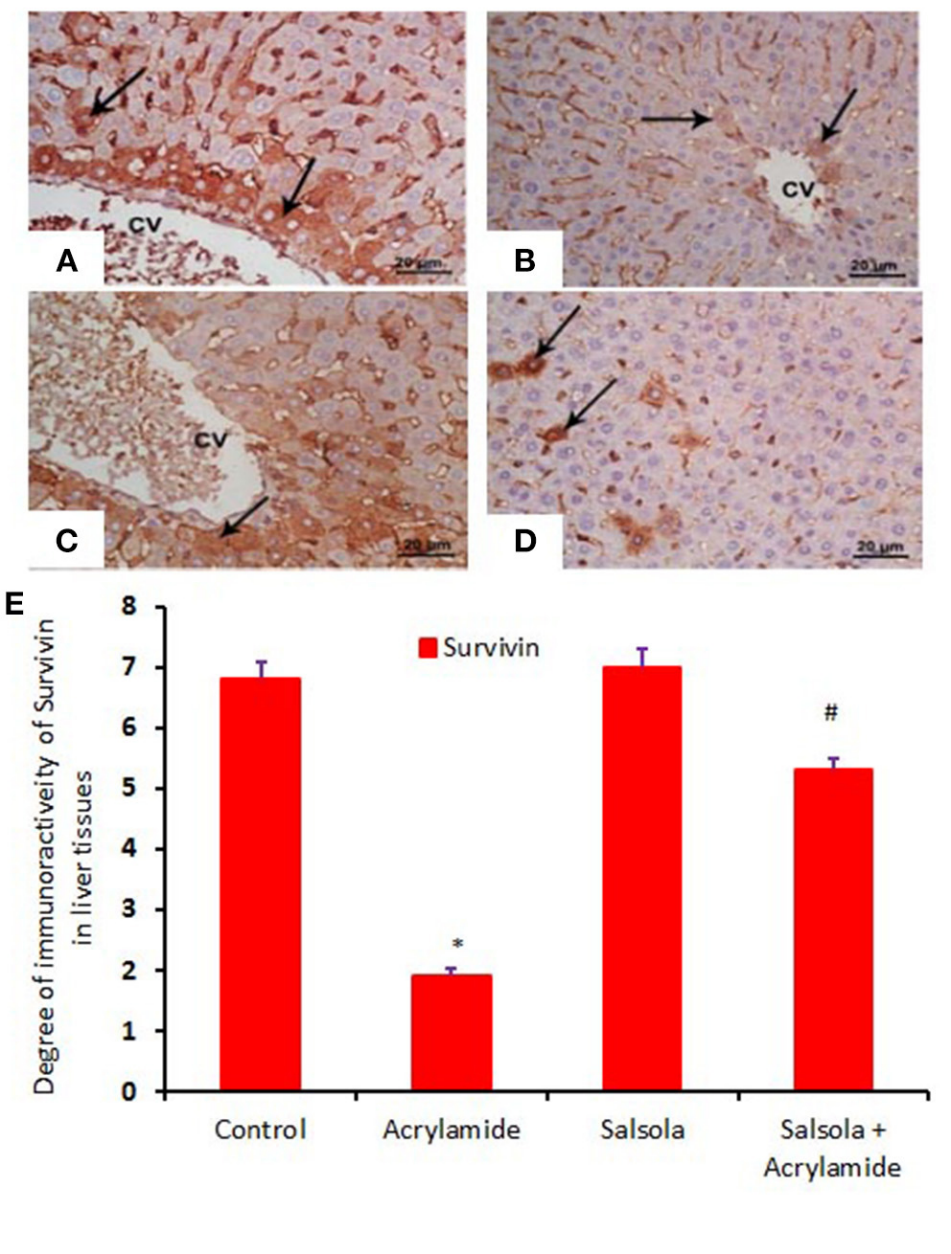
Photomicrographs of hepatic sections immunostained with survivin antibody, in the control group **(A)**, the *Salsola*-treated group **(C)**, the acrylamide-treated group **(B)**, and the group co-treated with *Salsola* and acrylamide **(D)**. The intensity of immunostaining was high in the control and *Salsola*-treated groups **(A,C)**, reduced in the acrylamide-treated group **(B)**, and restored to moderate levels in the co-treated rats **(D)**. Reactive liver cells (arrows) are usually next to central veins (CVs). Scale bar = 20 μm (original magnification = 200 ×). The degree of positive immunoreactivity for survivin is graphed in **(E)**. Densitometric values are statistically significant at *p < 0.05 vs. the control and *Salsola*-treated groups; ^#^*p* < 0.05 vs. the acrylamide-administered group.

When hepatic sections were immunostained for annexin, the intensity of immunostaining was high in the control and *Salsola*-extract groups (Figures 8A,C), reduced in the acrylamide-treated group (Figure 8B), and restored to moderate levels in the co-treated rats (Figure 8D). For the survivin immunoreactivity, the intensity of immunostaining was weak in the control and *Salsola* groups (Figures 9A,C), strong in the acrylamide-treated group (Figure 9B), and reduced to weak or moderate in the co-treated rats (Figure 9D). Reactive liver cells (arrows) are frequently seen next to central veins (CVs). The degrees of immunoreactivity for annexin and survivin are graphically represented in Figures 8E, 9E, respectively.

**Table 6:** Morphometric analysis and score lesions in the liver tissues of *Salsola imbricata extract* against acrylamide induced hepatic dysfunction in rats.

**Lesion**		**Control**	**Acylamide**	***Salsola extract***	**Salsola extract + Acrylamide**
Areas	Sinusoidal spaces	6.1 ± 0.2^a^	5.24 ± 0.3^a^	11.1 ± 2.2^c^	8.5 ± 2.02^b^
	Central veins	1 ± 0.04^a^	1.2± 0.03^a^	3.5 ± 0. 5^c^	1.2 ± 0.2^b^
	Portal blood vessels	1.8 ± 0.03^a^	1.6 ± 0.02^a^	4.1 ± 0.8^c^	2.7 ± 0.32^b^
Frequencies	Vacuolar and hydropic degeneration	0^c^	0^c^	21.2 ± 4.1^a^	10.5 ± 2.5^b^
	Inflammatory infiltrate	0^c^	0^c^	15 ± 3 ^a^	3 ± 0.3^b^
	Von Kupffer cell hyperplasia	0^c^	0^c^	9 ± 1.3 ^a^	4 ± 1.1^b^
	Single-cell Necrosis	0^c^	0^c^	16 ± 4.2 ^a^	6 ± 1.4^b^
Areas	Sinusoidal spaces	6.1 ± 0.2^a^	5.6 ± 0.3^a^	11.1 ± 2.2^c^	8.5 ± 2.02^b^
	Central veins	1 ± 0.04^a^	1.02 ± 0.03^a^	3.5 ± 0. 5^c^	1.2 ± 0.2^b^
	Portal blood vessels	1.8 ± 0.03^a^	1.68 ± 0.02^a^	4.1 ± 0.8^c^	2.7 ± 0.32^b^
Frequencies	Vacuolar and hydropic degeneration	0^c^	0^c^	29.2 ± 4.1^a^	10.5 ± 2.5^b^
	Inflammatory infiltrate	0^c^	0^c^	15 ± 3^a^	3 ± 0.3^b^
	Von Kupffer cell hyperplasia	0^c^	0^c^	9 ± 1.3^a^	4 ± 1.1^b^
	Single-cell Necrosis	0^c^	0^c^	16 ± 4.2^a^	6 ± 1.4^b^

*Values are mean ± SE for 4 slides/group. Means within the same row (in each parameter) carrying different superscripts are significantly different at p < 0.05*.

## Discussion

The current study showed that the tested *Salsola* extract significantly normalized the changes in liver biomarkers that occurred due to acrylamide-induced oxidative stress, represented by an increase in ALT, AST, GGT, total protein, and albumin levels. However, these effects were boosted by the antioxidant, and anti-apoptotic impacts of the *Salsola imbricata* extract. The histological and immunohistochemical results were reinforced and reflected the degree of hepatic damage caused by acrylamide.

The most likely mechanism that leads to the creation of acrylamide is the generation of acrolein, which is generated as a consequence of the thermal breakdown of glycerol, followed by the oxidation of acrolein with acrylic acid. Acrylic acid and acrolein may be formed from triglyceride breakdown products, which are generated during food frying (40, 41). The oxidative damage incurred by acrylamide is mostly associated with an increase in ROS production, as reported by others (42–44) and in this study. As known, lipid peroxidation due to oxidative stress is a cause of a decrease in antioxidant activity (45). This was confirmed by a depletion in the activity of SOD, GSH, and catalase and an increase in lipid peroxidation due to acrylamide administration. Reduced GSH disrupts the antioxidant defense system (46). Acrylamide induces organ and tissue redox impairments, especially in terms of mitochondrial DNA (47), while antioxidant enzymes counteract and reduce these redox impairments (48). Our findings, as well as others (42–44), show how acrylamide induces liver damage and stress through ROS generation, lipid peroxidation, and NO production. *Salsola imbricata* pre-administration increases the activities of liver antioxidants and reverses the effects of acrylamide intoxication.

Our findings show that the major components of the ethanolic extract of Salsola imbricata are variable types of flavonoids, in high concentrations such as catechin, Vanillic acid, Rutin catechol, myricetin, P hydroxyl benzoic acid and Syringic acid, while quercetin, resveratrol, o-Coumaric acid, and ellagic acid are present at moderate concentrations. All are implicated in reducing hepatic toxicity during health and disease (49, 50). Such confirmation was reported in this study and in others (51, 52). In parallel, the ethanolic extract of *Salsola imbricata* extract was rich in phenolic compounds such as vanillic acid, synergic acid, ellagic acid, and other related phenolic compounds that act either alone or in combination to protect the liver from toxicity induced by different chemical inducers, such as acrylamide and methotrexate (53–57).

Hepatic dysfunction is induced by acrylamide toxicity (58, 59). The toxicity of acrylamide is due to redox imbalance (60). The current study confirmed that acrylamide induces hepatic dysfunction, represented by the expression of genes related to apoptosis and necroptosis (61). Acrylamide induces liver apoptosis through the upregulation of Bax activation and the downregulation of Bcl2 expression. Significantly, a low degree of damage was reported in rats pre-administered *Salsola*.

Nrf2 regulates the expression of HO-1 and the defensive mechanisms of cells (62, 63). Nrf2 controls genes that are essential for the regulation of both oxidative stress and the antioxidant response. In investigations with Nrf2-knockout mice, a role for Nrf2 in the amelioration of oxidative stress has been suggested (64). Following *Salsola* treatment, the expression of Nrf2 and HO-1 is dramatically increased, indicating a critical functional role in the regulation of hepatic oxidative stress. The reported data (65) corroborate our findings, confirming the negative impacts of acrylamide on Nrf2/HO-1 signaling.

An imbalance between anti-inflammatory and proinflammatory cytokines causes inflammation, which is mediated by the alteration of inflammatory proteins (66). Acaroz et al. (67) found that several transcription factors, including NF-κB, can cause inflammation in response to proinflammatory cytokine (TNF-alpha and IL-1) activation. The activation of NF-κB increases inflammation and immunological responses and upregulates COX-2 activation (68–70). Acrylamide administration at a dose of 20 mg/kg of BW to experimental animals has been shown to considerably raise the levels of inflammatory cytokines such as TNF-α and IL-1 (1). The NF-κB gene plays a pivotal role in the expression of inflammatory mediators such as iNOS, which leads to NO production and activation, which can contribute to the nitrosative stress induced by acrylamide (71). It has been reported that the induction of the acute-phase response is accompanied by a decrease in IL-10 production, as reported by us and others (72).

The pre-administration of *Salsola imbricata* reduced acrylamide-induced liver inflammation by lowering inflammation-related markers, and increased anti-inflammatory cytokine production. TGF-β1 is the principal isoform reported in liver tissue. Its activation stimulates the phosphorylation of the downstream mediators Smad2/Smad3, which, in turn, stimulates the development of cardiac fibrosis (73). Acrylamide induced liver fibrosis and the upregulation of TGF-β1 expression, which was boosted by the pre-administration of *Salsola imbricata* extract. As a result, we might assume that the protective effects of *Salsola imbricata* extract on liver injuries are due, in part, to the inhibition of inflammation.

Bax and Bcl-2 are genes and members of the Bcl-2 family that influence cells' susceptibility to apoptosis (74). The Bcl-2 protein is an anti-apoptotic factor (75), while Bax is pro-apoptotic protein that increases during inflammation, chronic liver diseases, and fibrosis to promote apoptosis (76). Acrylamide administration increased Bax expression in the liver, leading to increased apoptosis. Conversely, Bcl-2 mRNA expression was downregulated by acrylamide. The prior administration of *Salsola imbricata* restored the changes in Bax and Bcl2, thereby preventing acrylamide-induced oxidative stress and apoptosis.

MAP kinase activation impacts hepatic Bcl-2 activity when the liver is subjected to stressors that cause apoptosis (77, 78). Hepatic programmed cell death may mediate hepatic toxicity (78, 79). The current study concluded that liver apoptosis was induced by acrylamide, which causes Bax, TGF-β1 and COX-2 activation, and decreases Bcl-2, Nrf2, HO-1, and antioxidant expression.

Annexins are a Ca^2+^-sensitive family of proteins that bind to negatively charged phospholipids and form unique interactions with other lipids and lipid microdomains. Annexins are involved in a wide range of intracellular functions, including the regulation of membrane dynamics, cell migration, proliferation, and death (80). Annexin can be utilized as a cell-surface protein to identify certain types of malignancies (81). The current study showed that acrylamide upregulated annexin immunoreactivity, while the pre-administration of the *Salsola imbricata* extract normalized such upregulation. By contrast, survivin (an inhibitor of apoptosis) was downregulated in acrylamide-administered rats and upregulated when *Salsola imbricata* extract was pre-administered to the acrylamide group. It has been reported that survivin is associated with liver cell regeneration and cell division (82), which coincides with our reported results.

## Conclusions

**Figure 10:**
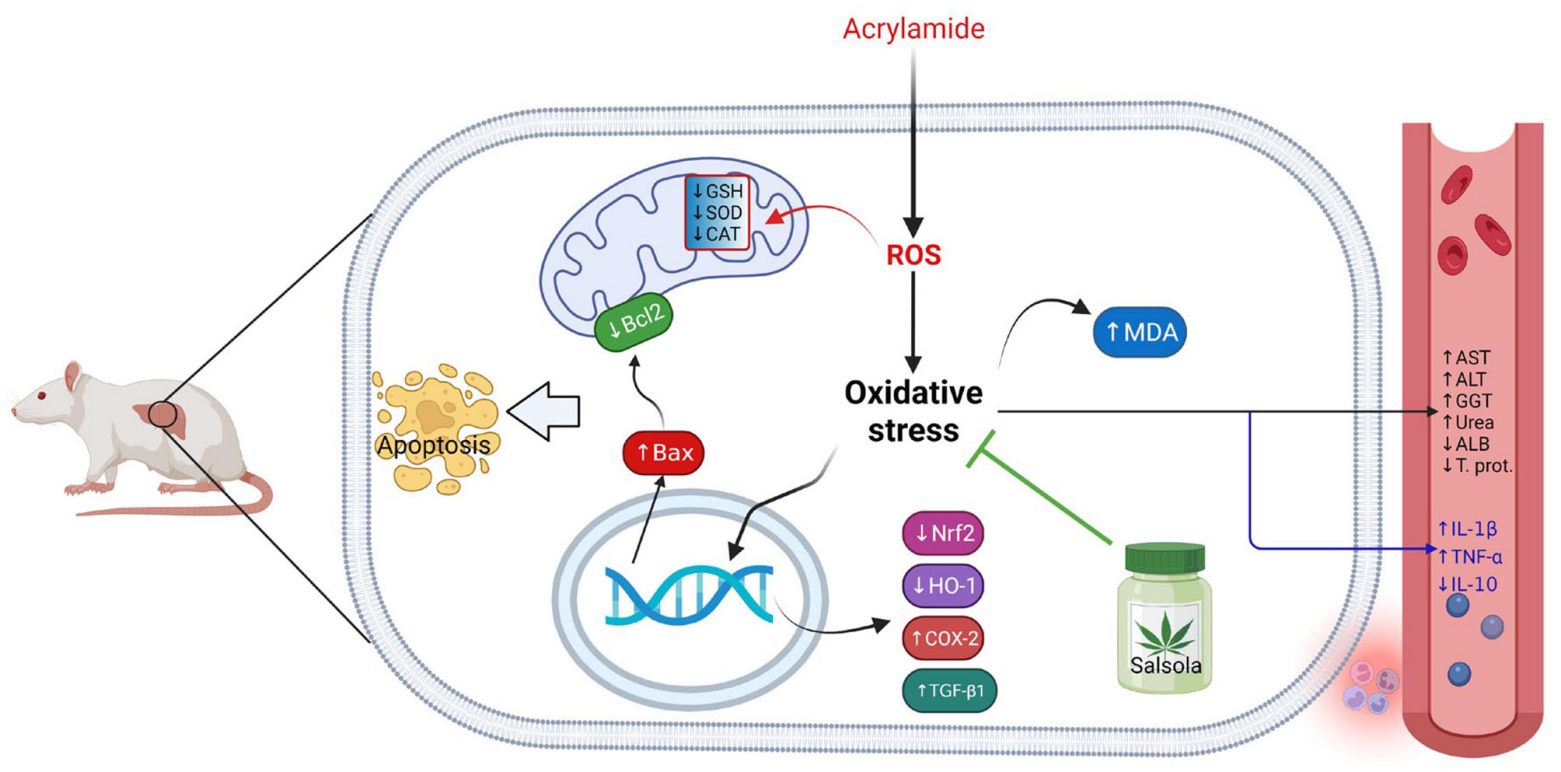
Graphical abstract represents the hepatoprotective impact of *Salsola imbricata* extract on acrylamide-induced inflammation, oxidative stress, and liver dysfunction.

The current study confirmed the ameliorative impact of *Salsola Imbricata* extract against the oxidative stress occurred due to acrylamide toxicity. The collective impacts of *Salsola Imbricata* extract against hepatic toxicity induced by acrylamide was summarized on Figure 10.

## Data Availability Statement

The original contributions presented in the study are included in the article/supplementary material, further inquiries can be directed to the corresponding author.

## Ethics Statement

The animal study protocol for this study was reviewed and approved by the ethical committee of Deanship of Scientific affairs, Taif University, Saudi Arabia, for the project number 136-441-1.

## Author Contributions

MS, AE-S, SS, and SA contributed data collection and analysis. MH, AA, GY, and FA revised data. MS and AE-S prepared the manuscript. MS, AE-S, and SS approved final gallery proof. All authors contributed to the article and approved the submitted version.

## Funding

The authors extend their appreciation to the deputyship for research and Innovation, Ministry of Education, in Saudi Arabia for funding this research work through project number 136-441-1.

## Conflict of Interest

The authors declare that the research was conducted in the absence of any commercial or financial relationships that could be construed as a potential conflict of interest.

## Publisher's Note

All claims expressed in this article are solely those of the authors and do not necessarily represent those of their affiliated organizations, or those of the publisher, the editors and the reviewers. Any product that may be evaluated in this article, or claim that may be made by its manufacturer, is not guaranteed or endorsed by the publisher.
